# The importance of test order in external and standardized test results: The case of PISA 2018

**DOI:** 10.1371/journal.pone.0309980

**Published:** 2024-09-09

**Authors:** R. van Grieken, J. D. Tena, Luis Pires, Ismael Sanz, Lilliana L. Avendaño-Miranda

**Affiliations:** 1 Chemical and Environmental Engineering Group, School of Experimental Sciences and Technology, Rey Juan Carlos University, Madrid, Spain; 2 Management School, University of Liverpool, Liverpool, United Kingdom; 3 Department of Economics, University of Sassari and CRENoS, Sassari, Italy; 4 Department of Applied Economics I, Faculty of Economics and Business, Rey Juan Carlos University, Madrid, Spain; 5 Social Policy Department, London School of Economics, London, United Kingdom; Institute of Medical Biochemistry Leopoldo de Meis (IBqM) - Federal University of Rio de Janeiro (UFRJ), BRAZIL

## Abstract

Standardized tests intend to reduce information asymmetry by providing a common and objective measure of students’ academic performance. The basic assumption underlying standardized testing is that differences in student performance on standardized tests should be attributed primarily to differences in the quality of education received by students. However, there is evidence that environmental factors can affect standardized test scores, which may result in anomalous observations or outliers that show a distortion of student performance. In this regard, the exclusion of Spain from PISA 2018 is particularly interesting as Spanish data met PISA 2018 Technical Standards but showed implausible student-response behavior. The aim of this paper is to complement the OECD’s analysis of Spain’s exclusion from PISA 2018 by exploring the potential reasons behind the outlier results, focusing on the Madrid region.

## 1. Introduction

Assessment is an essential practice in education. Educational assessment is of great interest to parents, teachers, educational institutions, government, and other decision-makers. School tests are designed for a variety of purposes. Standardized tests intend to reduce information asymmetry by providing a common and objective measure of students’ academic performance [1, 2]. Of relevance is the OECD’s PISA program, which assesses the reading, mathematics, and science literacy of secondary school students [3].

The basic presumption behind standardized testing is that “differences in the achievement of students on standardized tests should be primarily attributable to differences in the quality of education received by students” [4]. However, there is evidence that environmental factors may affect standardized tests results. For example, Ebenstein, Lavy and Roth [5] found that the students’ cognitive performance, during matriculation high school exams in Israel, was associated with air pollution level on the day of the test. Wen and Burke [6] obtained similar results linked to wildfire exposure in western United States, while Park Goodman, Hurwitz, and Smith [7] presented evidence of lower test results related to heat exposure in classrooms during the American PSAT exams. In other words, standardized testing results might be affected by environmental factors that may result in anomalous observations or outliers, showing a distortion of students’ performance. Outliers may be due to random variation that direct to misleading results, but they may also indicate a scientifically interesting event [8].

In 2018 PISA results for Spain were so atypical that they were excluded from the results report [3]. The exclusion of Spain from PISA 2018 is particularly interesting as Spanish data met PISA 2018 Technical Standards but showed implausible student-response behavior. The OECD released a report explaining the problems that caused the exclusion of Spain [9]. The aim of this paper is to complement the OECD’s analysis of Spain’s exclusion from PISA 2018 by exploring the potential reasons behind the outlier results, focusing on the Madrid region.

We chose Madrid region for three main reasons: 1) it is the region with the most abnormal deviation in Reading; 2) it represents a fourth of the Spanish Education System, and 3) Madrid conducted high-stakes exams for tenth-grade students which overlapped PISA testing. The coincidence of the testing period was one of the reasons that OECD pointed out as a possible cause of atypical results in Spain [9].

## 2. PISA: Characteristics, trends, and atypical results

The PISA survey releases comparative data on 15-years-old students in Reading, Mathematics, and Science every three years. In its last edition, 79 countries (37 belonging to the OECD and 42 associate countries) participated in PISA 2018. Each edition chooses one of the three evaluated competencies as the primary. This implies that there are more questions about the primary competence and that the OECD international report is more focused on it. More specifically, Reading was the primary competence in PISA 2018 (similar to PISA 2000 and 2006). The PISA survey provides rich information on students’ abilities in each competence and characteristics of schools and students.

PISA scores are decided based on the variation in results observed across all test participants. Thus, PISA uses item-response-theory models to describe the relationship between student proficiency, item difficulty and item discrimination. PISA defines a score of 500 as the average proficiency of students across OECD countries, with a standard deviation (a measure of variability) of 100 score points. Therefore, a one-point difference on the PISA scale corresponds to an effect size of 0.01. To interpret the meaning of students’ scores in substantive terms, recall that PISA scales are split into proficiency levels, defining the knowledge and skills needed to complete tasks successfully. Each proficiency level corresponds to a range of about 80 score points. Based on estimations on the average score-point difference across adjacent grades for countries, the OECD states that, on average across countries, the difference between adjacent grades is about 40 score points. However, the OECD reports refrain from expressing PISA score differences in terms of an exact "years-of-schooling" equivalent ([3], p. 43–44).

The results of the PISA assessments are estimates because they are obtained from samples of students using a limited set of assessment tasks. It publishes the confidence interval to determine when a difference is statistically significant. In doing it, PISA takes into account two sources of uncertainty, namely, the sampling error (around 2 to 3 PISA score points for most countries) and the measurement or imputation error (around 0.5 of a PISA score point in Reading, and 0.8 of a point in mathematics and Science). An additional source of uncertainty allows for comparison across different PISA assessments, based on the difference in the test instruments, items, and calibration samples, which results in a link error. For example, for comparisons between reading results in PISA 2018 and past PISA assessments, the link error corresponds to at least 3.5 score points ([3], p. 45).

One of the primary uses of the PISA reports is the comparative analysis of different education systems through time. Thus, each PISA edition shows the relative position of each country or region and policymakers aim to keep or improve that situation in subsequent years. The OECD General Secretary clearly states this: "PISA is not only the world’s most comprehensive and reliable indicator of students’ capabilities, it is also a powerful tool that countries and economies can use to fine-tune their education policies" ([3], p. 4). Moreover, in its PISA reports, the OECD analyses the performance evolution of participating countries and regions. It also highlights those with better results, such as the four participating Chinese regions in the last PISA edition (Beijing, Shanghai, Jiangsu and Zhejiang). These four regions have performed better than the rest of participating countries, except Singapore.

The 2007 evaluation of the impact of PISA reveals that “Countries that rank relatively high in PISA use the PISA results as a mechanism for evaluating their education system, but do not seem to have introduced any policy initiatives directly in light of PISA. On the contrary, in countries that perform relatively low, we identify a direct policy impact after the publication of the PISA results” ([10], p.8).

PISA highlights those countries with a positive trend evolution. This is the case of Estonia, which has become the European country with the best academic performance. In particular, after an improvement of 22 and 9 score points in Reading and Mathematics respectively, Estonia has reached the first position in the world PISA ranking in Reading (523 score points) while taking the third position in Mathematics (523 score points) after Japan and South Korea. The positive evolution of Portugal has also been remarkable. More specifically, despite being one of the most affected countries by the financial crisis in 2008, Portugal has increased their performance in Reading (22 score points), Mathematics (38 score points) and Science (33 score points). Other countries with even more significant improvement in PISA scores are Qatar, Poland, and Peru. From 2006 to 2018, Qatar has increased its PISA results in Reading (95 score points), Mathematics (96 score points) and Science (70 score points). Poland and Peru have experienced aggregate increases of more than 20 score points on average.

Of course, there are also countries with a negative trend. For example, since 2000, the evolution in Reading scores has been moderately negative in Australia, Finland, Iceland, and New Zealand, and sharply negative in South Korea, the Netherlands and Thailand (OECD, PISA 2018 Database, Table I.B1.10; Figure I.9.1). However, PISA performance remains relatively stationary in many other countries.

**Fig 1:**
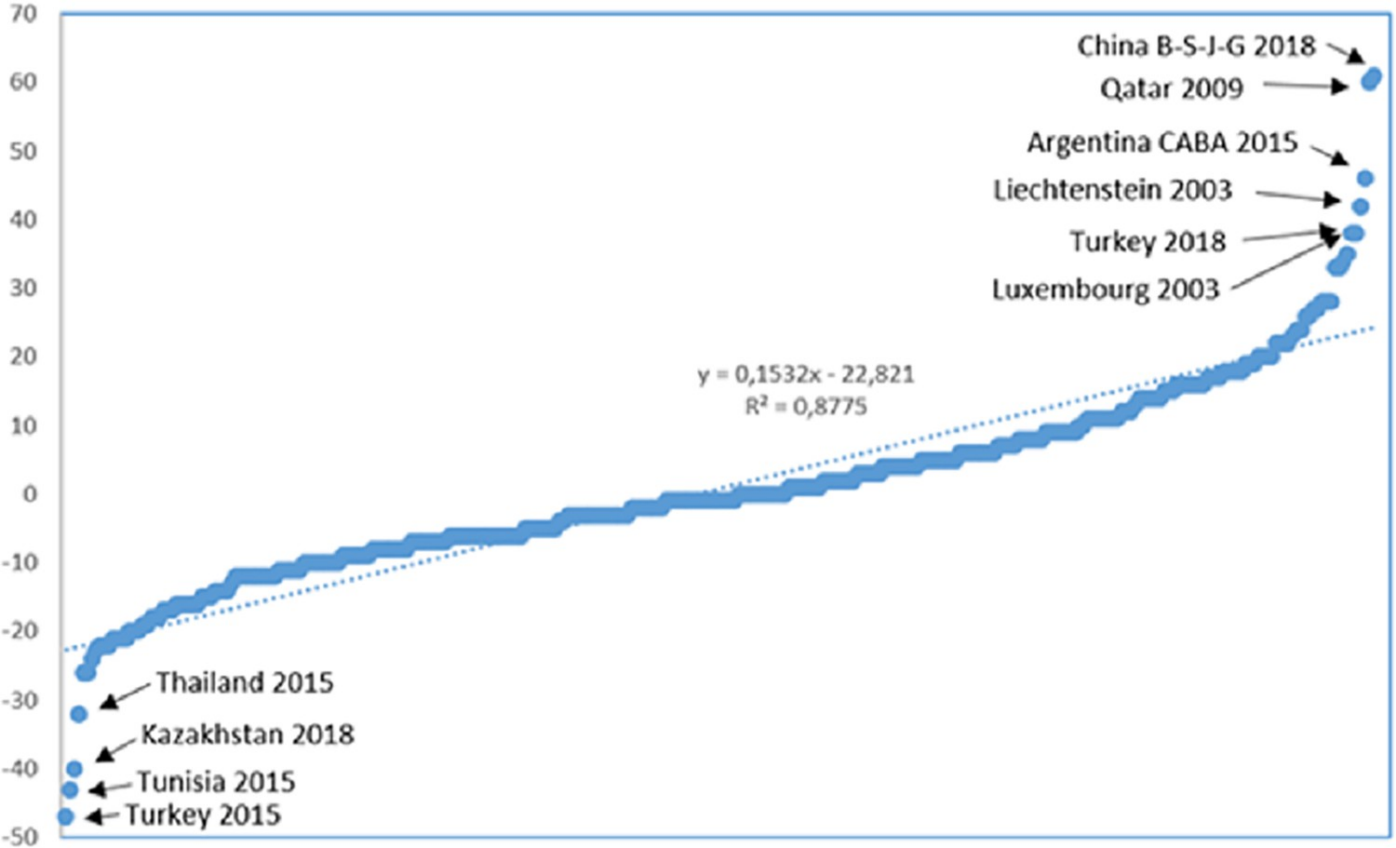
Change in score points between two consecutive PISA editions in Reading (2000–2018).

However, observing a sharp improvement or deterioration of a given country in the PISA ranking is rare. Fig 1 shows all the score changes in Reading between two PISA editions from 2000 to 2018. We observe that 87.3% and 60% of the 308 jurisdictions (countries or regions) are within the ±20 and ±10 interval, respectively. Furthermore, only 4.5% of the sample is outside the ±30 interval. Recall that 40 score points amount to the knowledge obtained in a whole academic year. Thus, it is unexpected that students in a given country gain or lose all the competencies they acquire in a year within three years. The most remarkable case is Turkey which in 2015 decreased by 47 score points in Reading (with 28 score points in Mathematics and 38 in Science). Still, it bounced back in 2018 with an increase of 38, 34 and 43 score points in Reading, Mathematics and Science, respectively. The OECD remarked on this anomalous situation, stating: "When considering results from all years, it is clear that PISA 2015 results–which were considerably lower–were anomalous, and neither the decline between 2012 and 2015, nor the recovery between 2015 and 2018, reflect the long-term trajectory" ([3], p. 340). Therefore, a change of 40 score points between two consecutive editions can be considered atypical, and the circumstances surrounding the implementation of the test could explain this anomaly, as we will discuss below.

To understand these results, it is worth noticing that the PISA test is very complex as its implementation requires many different people, companies, and education authorities. Any mistake in any of their steps could make results unreliable. For that reason, PISA has developed technical standards to set specific procedures for test implementation and data collection [11–15]. The test includes the following steps: elaboration of questions, translation of questions to the local language, photocopy of the test (if on paper) or saving it on tablets or computers (if online), choice and acceptance of the school and student sample, allocation of days for the test, correct application of the test by agents external to the school. A final fundamental element for a reliable interpretation of PISA results is that students will be focused on the questions. This is not a minor issue since about 7 out of 10 students informed putting less effort into the PISA test than they would have done if, for example, their performance on the test had counted towards their grades [3]. If a substantial number of students do not put in enough effort, this outcome will be reflected in the position of the region or country in the PISA ranking as in the case of Australia, whose mean performance has been declining over the period 2000–2018 [3].

Since 2000, PISA results have not been published on some occasions as they did not meet standards. In other circumstances, PISA results were issued, indicating not to use the information in international comparisons or study its trend. The OECD usually spots these problems after test results are analyzed. This is a complex process that lasts more than one year. The main OECD uses the following criteria to ensure the information is suitable: student- and school-level exclusions, minimum sample sizes, response rates, and inconsistencies and deviations from the expected patterns. Despite the complexity of the PISA evaluation, the OECD only had to exclude 11 out of 421 participant countries or regions in previous editions. However, there are relatively minor problems with a more significant number of cases where the OECD has decided they are statistically significant and comparable. Table 1 shows the 11 countries or regions excluded from PISA in some previous editions. There are two different cases. First, some are suspected of inflating their scores. This may be a credible strategy as the PISA test can be deemed the most prestigious evaluation of the educational system in a given country. This would be the case of Argentina, Kazakhstan, Malaysia, or Viet Nam which showed a massive improvement of their scores in the exclusion years and a return to the mean effect in subsequent years; the OECD excluded them from the PISA results due to these irregularities (3, 14) as we will describe below. Second, this group includes countries that experienced technical problems that did not drive scores in a particular direction, such as the Netherlands, United Kingdom, United States or Spain.

**Table 1:** PISA results in the excluded countries.

Country	Reading							Mathematics							Science							Difference with PREVIOUS edition*	Difference with NEXT edition*
	2000	2003	2006	2009	2012	2015	2018	2000	2003	2006	2009	2012	2015	2018	2000	2003	2006	2009	2012	2015	2018		
Netherlands		513	507	508	511	503	485		538	531	526	523	512	519		524	525	522	522	509	503		
United Kingdom	523	507	495	494	499	498	504	529	508	495	492	494	492	502	532		515	514	514	509	505	-21	-4
United States	504	495		500	498	497	505	493	483	474	487	481	470	478	499	491	489	502	497	496	502	-2	+6
Albania	349			385	394	405	405	381			377	394	413	437	376			391	397	427	417	+17	+19
Albania	349			385	394	405	405	381			377	394	413	437	376			391	397	427	417	+30	-10
Argentina	418					425	402	388					409	379	396					432	404		-28
Argentina CABA					429	475						418	456						425	475		+50	
Kazakhstan				390	393	427	387				405	432	460	423				400	425	456	397	+29	-59
Malaysia					398	431	415					421	446	440					420	443	438	+23	-5
Viet Nam					508	487	505					511	495	496					528	525	543	+18	
Spain	493	481	461	481	488	496	474	476	485	480	483	484	486	481	491	487	488	488	496	493	483	-22	

The year the exclusion occurred is colored

* In the main domain of the year of exclusion

Source: OCDE [3, 11–15].

Thus, for example, the Netherlands in PISA 2000 ([11], p. 186) and the United Kingdom in PISA 2003 ([12], p. 281) included this warning as school participation rates were exceptionally low. Similarly, Reading literacy results for the United States were excluded from the database and international reports because of an error in printing the test booklets in PISA 2006 ([12], p. 281). Albania’s data for parental occupation and school enrolment were deleted from the PISA 2012 international dataset due to evidence of systematic errors and violations of the PISA Technical Standards in the survey instruments, the procedures for test implementation and coding of student responses at the national level ([13], p. 283). For the same reasons, the PISA 2015 international database does not include all the information collected through student questionnaires for Albania. The OECD published this information in an additional dataset but forewarning that no attempt should be made to link the student data included in the international PISA database with the additional dataset for Albania ([14], p. 269). The international dataset did not include Argentina in PISA 2015, but this information is available as a separate dataset. Still, Ciudad Autónoma de Buenos Aires (CABA) data were fully included in the international dataset even though the national defined target population deviated significantly from the desired target population ([14], pp. 269–270). When assessing the results of the PISA test, all multiple-choice questions and certain short-answer questions are automatically scored within the system. On the contrary, open-ended questions that do not conform to this scoring are evaluated and scored by experts. It was discovered that scores for human-scored items submitted by Kazakhstan were contradictory with the success rates in preceding PISA cycles and were virtually unrelated to scores on multiple choice items. Thus, data for Kazakhstan in PISA 2015 were removed from the international dataset (but available as a separate dataset) because of leniency among national experts, which forced the elimination of all human-scored items ([14], p. 271). Data for Malaysia in PISA 2015 are included in a separate database because of a low response rate among the initially sampled schools ([14], p. 271). In the last PISA 2018, the international dataset did not include financial literacy sample data for the Netherlands because of a low school participation rate ([15], pp. 9–10). Data for Viet Nam in PISA 2018 were removed from the international dataset (available as a separate dataset) because of several minor violations of implementation standards ([15], p. 12). Finally, PISA 2018 reading results for Spain were not published in the results report [3] and are not included in OECD average results but are available as a separate dataset [9].

## 3. PISA in Spain and Madrid region

Spain has participated in PISA since its creation, PISA 2000. Although the central government sets general Education Laws in Spain, autonomous regions oversee their implementation and development. In Spain there are 17 autonomous regions and 2 autonomous cities (Ceuta and Melilla). For this reason, since PISA 2003, autonomous regions have been gradually incorporated into PISA, making their results comparable to other participant countries. Table 2 reports the PISA score of the different autonomous regions and cities. The anomalous score reduction in PISA 2006 (mainly due to Reading) and the subsequent recovery in PISA 2009. A similar situation can be observed for the autonomous region of Murcia, with a decrease of 18 score points in PISA 2012 and a subsequent increase of 24 score points in PISA 2015. We can observe a sustained improvement in the majority of other regions.

**Table 2:** PISA results in Spanish regions (2000–2018).

READING	PISA Results							Difference with previous year					
	2018	2015	2012	2009	2006	2003	2000	2018	2015	2012	2009	2006	2003
Andalusia	466	479	477	461	445			-13	2	16	16		
Aragon	490	506	493	495	483			-16	13	-2	12		
Asturias	495	498	504	490	477			-3	-6	14	13		
Balearic Islands	479	485	476	457				-6	9	19			
Basque Country	475	491	498	494	487	497		-16	-7	4	7	-10	
Canary Islands	472	483		448				-11					
Cantabria	483	501	485	488	475			-18	16	-3	13		
Castile and Leon	497	522	505	503	478	499		-25	17	2	25	-21	
Castile-La Mancha	478	499						-21					
Catalonia	484	500	501	498	477	483		-16	-1	3	21	-6	
Ceuta	404												
Comunidad Valenciana	473	499						-26					
Extremadura	464	475	457					-11	18				
Galicia	494	509	499	486	479			-15	10	13	7		
La Rioja	467	491	490	498	492			-24	1	-8	6		
Madrid	474	520	511	503				-46	9	8			
Melilla	438												
Murcia	481	486	462	480				-5	24	-18			
Navarre	472	514	509	497	481			-42	5	12	16		
SPAIN	477	496	488	481	461	481	493	-19	8	7	20	-20	-12

Source: OCDE [3, 11–15].

However, PISA 2018 is especially atypical. Such a situation has produced the exclusion of Spain and all its autonomous regions. All of them worsen score points, and the magnitude of the deterioration is highly unreliable in some cases. The most affected autonomous regions were Madrid (-46), Navarre (-42), Comunidad Valenciana (-26), Castile and Leon (-25) and La Rioja (-24). Moreover, the case of Madrid is especially remarkable because it was in the highest rank, and its situation drastically changed in PISA 2018.

Interestingly, with minor exceptions for some specific schools, Spain did not find any significant problem applying the PISA test before PISA 2018. Thus, the exclusion of Spain from PISA 2018 is particularly interesting as Spanish data met PISA 2018 Technical Standards but showed implausible student-response behavior. According to the OECD, around 68% students across OECD countries put less effort on the PISA test than they would have done if the test had counted in their grades. Therefore, the main problem was the large number of students who acknowledged “having spent very little effort on the PISA test they just completed” and hence did not achieve their expected scores on the reading test ([9], p. 8). The accuracy or reliability of answers is a common problem in many surveys. However, it was the first time that this problem caused the exclusion of a country from the PISA ranking. Although some members of the education system (teachers, principals, parents, students) opposed these tests, this is not a significant problem for their implementation as these schools are substituted or removed from the sample. However, there was no example of a general lack of interest by participant students that caused the exclusion of a country so far. As a result, the OECD released an eight-page report, in the form of an appendix, explaining the results of a study on the application of PISA 2018 in Spain that caused its exclusion [9]. In the other 10 cases of exclusion, there was only a short explanation consisting of a few paragraphs in the Technical Report.

To complement the analysis of the OECD about the exclusion of Spain from PISA 2018, in the following sections, we show a statistical analysis of the possible causes of abnormal results in the region of Madrid. Three main reasons explain the choice of Madrid. Firstly, it is the region with the most abnormal deviation in Reading. Secondly, it is highly representative of the country because it is a large region that approximatively represents a fourth of the Spanish Education System. Thirdly, the OECD pointed out as a possible reason of the lack of motivation of some of the Spanish students that "in 2018, some regions in Spain conducted their high-stakes exams for tenth-grade students earlier in the year than in the past, which resulted in the testing period for these exams coinciding with the end of the PISA testing window" [9]. As shown in Table 3, Madrid was one of the regions where different schools differed about when they set their school exams. This allows for identifying the importance of this event on PISA scores. Moreover, the PISA test overlapped in some cases with another external test at the regional level for tenth-grade students. Due to these reasons, the Madrid region is, in principle, an interesting example of how crowded exam schedules affect students’ performance.

**Table 3:** Calendar of school exams and external tests of tenth-grade students in Madrid 2018.

PISA application week		LOMCE Test	Third evaluation exams
1	2–6 April		
2	9–13 April		
3	16–20 April		
4	23–27 April	24–25 April	
5	30 April-4 May		
6	7–11 May		
7	14–18 May		
8	21–25 May		22–25 May
9	28 May-1 June		28–29 May
10	4.8 June		

Source: OCDE [9].

## 4. Data and methodology

The dataset is built from all plausible values for Spain in 2018 PISA report that were published in December 2019 as explained in Annex A9 (2020a) [9].

As we discussed previously, the 2018 PISA results could be affected by two events in the Madrid region: 1) participation in the regional external and standardised test that all students in their final year of compulsory school must take (LOMCE test); and 2) the 2017/18 academic calendar change for the third evaluation in the region (Table 3). In both cases, their impact is estimated using a difference-in-difference approach. For the first event, the regional test affected student performance at PISA because the PISA test took place from April 15^th^ to May 30^th,^ while the regional test came about on April 26^th^-27^th^. To identify the effect of this event, we take advantage of the fact that our control group, grade repeater students, only take the PISA but not the regional assessment. In contrast, non-repeaters (treatment group) take both exams. Thus, we estimate the impact on PISA score of taking the PISA test after the regional test or in the exam week in May affects students’ scores compared to a control group of students for whom PISA do not overlap either with the regional test nor the exam period. In the specification we include information of individual, family and school characteristics that could control for this fact. Thus, the implicit assumption in our approach is that, once we control for these characteristics, performance differences are entirely explained by the action of treatment.

We aim to account for the effect of two different explanations for the extreme results in Madrid regions, namely the clash with the regional test and the school evaluation. For this purpose, we employ two difference in difference specifications. The first one compares non-repeater students who take the PISA test after the regional test (first difference) with a control group of repeater students, who do not take the PISA test in any case. The second specification compares students in non fee-paying schools when the PISA clashes and when it does not clash with the exam period (first difference) with a control group of students in fee paying schools that are not affected by the overlapping of the two tests (second difference). Note that while a difference in difference approach allows treatment and control groups to be different, the implicit assumption is that these differences are accentuated by the effect of treatment. For example, repeaters and non-repeaters students get different scores in the PISA test under the null hypothesis and the approach tests if the difference between the two groups of students increases or decreases when a non-repeater student takes both the regional and the PISA tests. Descriptive statistics of the variables employed in the difference-in-difference models are shown in S1 Appendix.

Based on the discussion in the previous paragraph, the model in the first specification can be defined as follows:

$$Y_{i}=\beta_{0}+\beta_{1}D+\beta_{2}NR+\beta_{3}(D*NR)+\sum_{k=1}^{K}\gamma_{k}X_{i}+\varepsilon_{i},$$ (1)

where *Y*_*i*_ is the dependent variable that represents the relevant 2018 PISA score by student *i*; D is a dummy variable that takes value 1 if the PISA test took place after the regional test, and 0 otherwise; *NR* takes value 1 for non-repeater students and 0 otherwise; *X*_*i*_ is the ith observed student’s characteristic; *β*_0_ to *β*_3_ and *γ*_*k*_ for *k* = 1 to *K* are parameters to be estimated; and *ε*_*i*_, is an error component. In particular, *β*_0_ is the intercept, *β*_1_ and *β*_2_ represent the impact on the 2018 PISA score of having the PISA test after the regional test and being a non-repeater student, respectively. Our focus parameter *β*_3_ reflects the joint impact of being a non-repeater student and having the PISA test after the regional test. This is a fundamental consideration as only non-repeater students take both the PISA and the regional tests. The *K* parameters *γ*_*k*_ indicate the influence of the individual covariates on the response variables. Model (1) and all the subsequent specifications are estimated by OLS.

For the second case, we follow a similar strategy to estimate the impact of the 2017/18 academic calendar change in the region on PISA outcome. Under the new calendar, the 2018 PISA clashes with the exam period in non-fee paying schools of the Madrid region by the end of May. Fee-paying schools are the control group in this case. Accordingly, we estimate the following model:

$$Y_{i}=\beta_{0}^{\prime }+\beta_{1}^{\prime }ME+\beta_{2}^{\prime }NF+\beta_{3}^{\prime }(ME*NF)+\sum_{k=1}^{K}{\gamma_{k}\prime X}_{i}+\varepsilon_{i,}^{\prime },$$ (2)

where variables are similarly defined to expression (1) except for *ME* which takes value 1 if the 2018 PISA exam happens in May and 0 otherwise, and *NF* is a dummy variable taking value 1 for non-fee paying schools and 0 otherwise.

The estimation of the previous two models will provide a clear picture of the effect of two different scheduled events on students’ academic performance. More specifically, our focus parameter in specification (1) is *β*_3_ that indicates the expected score difference of students who took the PISA test after the regional test compared to those who did not take that exam once we control for observed students’ characteristics. In specification (2), our focus parameter is *β*_3_′, that indicates the expected score reduction from taking a PISA exam while preparing their school evaluation once we control for observable student characteristics.

## 5. Empirical results and discussion

We start the empirical analysis by estimating the importance of different individual and institutional determinants of plausible PISA scores using regression analysis in the region of Madrid. Table 4 shows the estimation results for specification (2) in the previous Section. Estimation results are generally consistent with initial expectations. The only exception is the number of minutes devoted to different abilities that negatively impact performance. A plausible explanation for this is that a potential reverse causality problem makes this estimation biased, i.e., students who have lagged in these subjects need more study time to make up work. This observation is consistent with Kuehn and Landeras [16] that suggests an endogeneity bias in a similar estimation. To tackle this problem, they propose a two-least square (2LS henceforth) estimation with homework time and time spent in private lessons studying math as the proposed instruments for science study time. However, these instruments are also likely to be affected by students’ performance, which would invalidate the exclusion restriction assumption. Therefore, we propose using the average number of teaching hours in that subject and the total number of students in that school. Although no instrumental variable is free of criticism, decisions about teaching hours at the school level are likely to affect individuals’ habits while not directly guided by individual mark expectations. The effect of minutes devoted to the different subjects is no longer negative in the 2LS regression, while the impact of all the other variables is qualitatively similar.

**Table 4:** Determinants of PISA scores.

DEPENDENT VARIABLE	READING	MATHEMATICS	SCIENCE	GLOBAL COMPETENCIES
female	10.61***	-19.61***	-15.07***	10.93**
	(3.431)	(3.300)	(3.639)	(4.280)
age	8.493	7.101	1.234	4.011
	(5.240)	(4.787)	(4.884)	(6.961)
inmigrant	-17.59***	-18.87***	-10.33	-13.50
	(6.581)	(6.371)	(7.608)	(10.84)
motherinmigrant	7.614	0.0521	4.729	7.253
	(5.235)	(5.889)	(5.147)	(6.759)
foreignlanguage	-5.842	-2.991	-10.06	-5.915
	(6.116)	(7.087)	(8.142)	(11.58)
minutsread	-0.0929***			
	(0.0233)			
ESCS	10.92***	15.06***	11.83***	9.516*
	(3.849)	(4.383)	(4.324)	(5.498)
biling	18.73**	9.153	15.19**	7.842
	(7.883)	(6.712)	(6.341)	(7.930)
nobiling	1.091	-0.469	0.913	-7.709
	(10.72)	(7.765)	(7.263)	(10.88)
private	8.454	8.885	4.851	13.29
	(12.24)	(10.22)	(10.10)	(11.16)
charternobiling	0.967	-5.397	-3.109	-4.770
	(6.244)	(5.491)	(5.375)	(6.997)
ESCSschool	13.49	18.04	8.586	15.25
	(16.76)	(11.91)	(12.44)	(15.35)
ESCSschool2	5.598	0.549	4.377	-4.035
	(17.43)	(14.14)	(14.45)	(16.34)
ESCS2	-1.193	-2.017	-1.339	1.226
	(2.188)	(2.687)	(2.564)	(3.159)
DATMadrid	33.44***	19.50**	20.95**	27.28***
	(11.00)	(8.011)	(8.261)	(9.747)
DATEast	30.14***	18.28**	22.42**	20.67*
	(11.35)	(8.908)	(9.172)	(10.55)
DATSouth	3.666	21.02**	15.52*	14.17
	(12.42)	(8.343)	(8.791)	(10.38)
DATNorth	33.94***	23.88***	23.30***	28.36**
	(12.25)	(8.872)	(8.790)	(11.80)
norepeaters	66.46***	77.75***	74.17***	74.15***
	(4.118)	(4.547)	(3.855)	(5.348)
aftermadridtest	-11.36	-6.672	-8.444	-11.79
	(8.056)	(5.610)	(6.932)	(7.991)
weektestmay	-9.091	-0.0203	0.911	1.199
	(6.323)	(4.445)	(4.311)	(5.774)
minutsmath		-0.0727***		
		(0.0208)		
minutscie			0.0867***	
			(0.0118)	
minutstotal				-0.00615
				(0.00561)
Constant	295.4***	332.9***	391.6***	406.7***
	(84.71)	(76.81)	(76.96)	(109.5)
Observations	12,264	12,264	12,075	10,092
R-squared	0.290	0.340	0.279	0.243

Standard errors between parentheses.

*** p<0.01

** p<0.05

* p<0.10

Regardless of the estimation method, girls get higher scores than boys in Reading and Global competencies. However, girls get lower scores than boys in Mathematics and Science. Students who repeated the course reduced their expected score by at least 70 marks compared to non-repeaters. Younger students who have not repeated the course get better results. The expected scores of immigrants are generally lower. However, it is remarkable that, after controlling for the migrant status of the student, having an immigrant mother or a mother who does not speak Spanish does not affect the student’s score. The ESCS index positively affects PISA performance, especially in math, but the squared value of this variable is not significant.

When we turn our attention to the type of school, private schools, which is the reference variable, get the best results followed (in this order) by bilingual private schools, bilingual charter schools, non-bilingual charter schools and non-bilingual IES.

Looking at different geographical areas, compared with the reference case (DAT west) we observe that students in DAT west schools get significantly lower expected scores for all competencies. West area is the richest area and the one where the best results were expected. This negative effect is more prominent in Reading (about 30 marks lower). South DAT only negatively affects Reading (about 32 marks). From these results, it is highly remarkable that DAT west shows a significant impact on PISA scores even after controlling for many different student and school characteristics in the regression analysis. It could indicate that some unobserved factors associated with the test’s application could have affected students in this area, for example, lack of engagement or experience of applicators.

Taking the PISA test after the regional test in Madrid negatively impacted performance. However, the effect is not significant at the conventional values. Moreover, the correlation of PISA scores with exam week in May is almost negligible. Overall, these results do not suggest that other exams could explain the deficient performance of Madrid students in PISA. Not all students and schools were similarly affected by these two events. Only non-repeater students would be affected by the overlap of the PISA and Madrid tests. Thus, adding the interaction term between non-repeaters and after the Madrid test to the regression analysis previously reported in Table 5 we would estimate how this affects the student’s expected score (which corresponds to specification (1) in the previous section). In this estimation, repeater students who took the PISA test after the Madrid test would be the control group.

**Table 5:** Difference in difference estimation for the impact of the PISA and Madrid test overlap on non-repeater students.

	(1)	(2)	(3)	(4)
VARIABLES	READING	MATHEMATICS	SCIENCE	GLOBAL COMPETENCIES
female	10.61***	-19.61***	-15.05***	10.92**
	(3.431)	(3.305)	(3.640)	(4.304)
age	8.492	7.119	1.145	4.045
	(5.214)	(4.797)	(4.863)	(6.912)
inmigrant	-17.59***	-18.89***	-10.25	-13.51
	(6.586)	(6.355)	(7.591)	(10.84)
motherinmigrant	7.613	0.0667	4.637	7.271
	(5.257)	(5.900)	(5.184)	(6.787)
foreignlanguage	-5.842	-2.975	-10.11	-5.875
	(6.066)	(7.063)	(8.103)	(11.51)
minutsread	-0.0929***			
	(0.0234)			
ESCS	10.93***	15.03***	11.93***	9.492*
	(3.861)	(4.414)	(4.356)	(5.529)
biling	18.73**	9.158	15.16**	7.852
	(7.886)	(6.719)	(6.324)	(7.928)
nobiling	1.091	-0.480	0.966	-7.706
	(10.73)	(7.775)	(7.273)	(10.88)
private	8.457	8.822	5.151	13.21
	(12.20)	(10.20)	(10.05)	(11.16)
charternobiling	0.967	-5.389	-3.146	-4.760
	(6.236)	(5.490)	(5.351)	(6.984)
ESCSschool	13.49	17.93	9.072	15.19
	(16.69)	(11.89)	(12.38)	(15.32)
ESCSschool2	5.594	0.650	3.899	-3.950
	(17.40)	(14.15)	(14.35)	(16.37)
ESCS2	-1.194	-1.998	-1.431	1.246
	(2.219)	(2.734)	(2.602)	(3.201)
DATMadrid	33.44***	19.46**	21.11**	27.25***
	(10.97)	(7.971)	(8.209)	(9.699)
DATEast	30.15***	18.27**	22.50**	20.67*
	(11.35)	(8.892)	(9.147)	(10.56)
DATSouth	3.666	21.03**	15.52*	14.19
	(12.42)	(8.349)	(8.780)	(10.41)
DATNorth	33.94***	23.89***	23.27***	28.36**
	(12.25)	(8.874)	(8.811)	(11.79)
norepeaters	66.50***	76.84***	78.43***	73.03***
	(8.136)	(8.834)	(8.016)	(9.176)
aftermadridtest	-11.33	-7.495	-4.551	-12.84
	(10.11)	(8.409)	(8.445)	(10.60)
weektestmay	-9.091	-0.0186	0.900	1.203
	(6.328)	(4.450)	(4.311)	(5.777)
norepeatersaftertest	-0.0499	1.134	-5.360	1.414
	(8.586)	(9.051)	(8.803)	(10.21)
minutsmath		-0.0727***		
		(0.0207)		
minutscie			0.0866***	
			(0.0119)	
minutstotal				-0.00612
				(0.00560)
Constantv	295.4***	333.3***	389.9***	407.0***
	(85.38)	(76.93)	(77.66)	(109.9)
Observations	12,264	12,264	12,075	10,092
R-squared	0.290	0.340	0.279	0.243

Standard errors between parentheses.

*** p<0.01

** p<0.05

* p<0.10

Similarly, another possible event that could explain a negative score in the PISA test is its overlap with the exam week in May. However, only students in public and charter schools take these exams while those in private schools take their exams in May. Thus, adding the interaction term between exam week in May and private school students to the baseline regression 2 would identify the impact of this event. Therefore, a regression model with the interaction effect just described is a difference in difference estimation where the students in the control and treatment groups are those who took the PISA test in May and belonged to a non-fee paying (public and charter) and private school, respectively.

Tables 5 and 6 show estimates of the strategies explained in the previous paragraph. For the sake of simplicity, only foci parameters are reported. Consistently with logit intuition, private school students who are non-affected by May exams perform better than their counterparts in non-fee paying schools. However, the effect is not significant at the conventional values. On the other hand, the overlap of the PISA and Madrid proof does not explain the different performance results in repeater and non-repeater students. Overall, these results suggest that a crowded schedule due to overlapping with other tests or exams did not significantly impact students affected by these events.

**Table 6:** Difference in difference estimation for the impact of the PISA and week exam in May overlap on non-repeater students.

	(1)	(2)	(3)	(4)
VARIABLES	READING	MATHEMATICS	SCIENCE	GLOBAL COMPETENCIES
female	10.64***	-19.60***	-15.04***	11.07***
	(3.428)	(3.294)	(3.636)	(4.282)
age	8.485	7.099	1.237	4.020
	(5.245)	(4.789)	(4.892)	(6.967)
inmigrant	-17.58***	-18.87***	-10.32	-13.48
	(6.574)	(6.368)	(7.605)	(10.83)
motherinmigrant	7.654	0.0655	4.761	7.263
	(5.267)	(5.892)	(5.163)	(6.765)
foreignlanguage	-5.834	-2.990	-10.05	-5.842
	(6.128)	(7.084)	(8.137)	(11.59)
minutsread	-0.0936***			
	(0.0231)			
ESCS	10.97***	15.07***	11.87***	9.621*
	(3.858)	(4.382)	(4.328)	(5.504)
biling	19.00**	9.248	15.43**	8.343
	(7.988)	(6.798)	(6.401)	(7.991)
nobiling	1.209	-0.426	1.024	-7.456
	(10.70)	(7.776)	(7.257)	(10.87)
private	11.22	9.849	7.213	18.32
	(14.50)	(12.31)	(11.20)	(12.60)
charternobiling	1.032	-5.374	-3.043	-4.623
	(6.247)	(5.493)	(5.378)	(7.001)
ESCSschool	13.68	18.10	8.741	15.57
	(17.02)	(12.05)	(12.53)	(15.47)
ESCSschool2	5.564	0.536	4.372	-4.044
	(17.76)	(14.28)	(14.54)	(16.51)
ESCS2	-1.221	-2.025	-1.358	1.160
	(2.195)	(2.684)	(2.570)	(3.165)
DATMadrid	33.79***	19.62**	21.25**	27.91***
	(11.31)	(8.116)	(8.332)	(9.866)
DATEast	30.42***	18.38**	22.66**	21.16**
	(11.41)	(8.894)	(9.154)	(10.52)
DATSouth	3.739	21.05**	15.59*	14.28
	(12.57)	(8.412)	(8.886)	(10.47)
DATNorth	33.68***	23.79***	23.08***	27.99**
	(12.40)	(9.060)	(8.903)	(11.99)
norepeaters	66.44***	77.74***	74.17***	74.12***
	(4.110)	(4.551)	(3.852)	(5.346)
aftermadridtest	-11.55	-6.737	-8.609	-12.18
	(8.055)	(5.600)	(6.934)	(8.035)
weektestmay	-13.85	-1.672	-3.177	-7.363
	(15.86)	(11.17)	(8.860)	(12.47)
weektestmaynoprivate	5.458	1.894	4.690	9.905
	(17.12)	(12.04)	(10.20)	(13.92)
minutsmath		-0.0728***		
		(0.0207)		
minutscie			0.0864***	
			(0.0119)	
minutstotal				-0.00618
				(0.00562)
Constant	295.1***	332.8***	391.2***	405.6***
	(85.00)	(77.04)	(77.24)	(109.8)
Observations	12,264	12,264	12,075	10,092
R-squared	0.290	0.340	0.279	0.244

Standard errors between parentheses.

*** p<0.01

** p<0.05

* p<0.10

Knowing that PISA asks school principals to complete a questionnaire covering the school system and school characteristics [14], the timing of the PISA test is not randomly allocated but depends on schools’ requirements. Thus, it is possible that school principals and managers in centers that take the PISA tests during the first weeks could be more competent to envisage potential risks associated with a crowded schedule. This could affect the validity of our estimation results if students’ performance in PISA is also correlated with the ability of schools’ principals. To tackle this concern, we perform the previous regression analyses with an estimation sample that only considers students who took the PISA test within two weeks of the Madrid tests. Table 7 shows the results of this estimation. As our analysis does not show any significant results, we believe that the reason may be a combination of different events that cannot be estimated, for example, the quality and experience of the applicators, students negatively disposed towards the PISA test, students’ well-being. Furthermore, while a biased significant result is a common concern in empirical studies, results in our analysis are not significant.

**Table 7:** Difference in difference estimation for the impact of the PISA test within two weeks of the Madrid tests.

	(1)	(2)	(3)	(4)
VARIABLES	READING	MATHEMATICS	SCIENCE	GLOBAL COMPETENCIES
female	10.64***	-19.60***	-15.03***	11.06**
	(3.428)	(3.298)	(3.637)	(4.303)
age	8.488	7.119	1.151	4.061
	(5.220)	(4.800)	(4.871)	(6.919)
inmigrant	-17.58***	-18.89***	-10.25	-13.49
	(6.582)	(6.353)	(7.591)	(10.84)
motherinmigrant	7.656	0.0815	4.670	7.285
	(5.292)	(5.903)	(5.201)	(6.793)
foreignlanguage	-5.832	-2.973	-10.10	-5.793
	(6.078)	(7.061)	(8.098)	(11.52)
minutsread	-0.0936***			
	(0.0232)			
ESCS	10.97***	15.05***	11.96***	9.593*
	(3.867)	(4.411)	(4.358)	(5.532)
biling	19.00**	9.256	15.38**	8.359
	(7.992)	(6.807)	(6.382)	(7.988)
nobiling	1.207	-0.437	1.068	-7.451
	(10.71)	(7.784)	(7.265)	(10.86)
private	11.22	9.815	7.358	18.26
	(14.48)	(12.29)	(11.15)	(12.59)
charternobiling	1.033	-5.365	-3.083	-4.611
	(6.239)	(5.492)	(5.351)	(6.988)
ESCSschool	13.67	18.00	9.203	15.49
	(16.96)	(12.03)	(12.47)	(15.45)
ESCSschool2	5.577	0.644	3.909	-3.941
	(17.74)	(14.29)	(14.45)	(16.54)
ESCS2	-1.218	-2.005	-1.447	1.184
	(2.223)	(2.730)	(2.606)	(3.205)
DATMadrid	33.79***	19.58**	21.38***	27.87***
	(11.28)	(8.077)	(8.286)	(9.819)
DATEast	30.42***	18.36**	22.72**	21.16**
	(11.41)	(8.879)	(9.137)	(10.54)
DATSouth	3.739	21.05**	15.59*	14.30
	(12.57)	(8.418)	(8.876)	(10.50)
DATNorth	33.68***	23.79***	23.07***	27.99**
	(12.40)	(9.060)	(8.917)	(11.97)
norepeaters	66.32***	76.78***	78.29***	72.77***
	(8.151)	(8.890)	(8.020)	(9.210)
aftermadridtest	-11.66	-7.616	-4.828	-13.45
	(10.17)	(8.464)	(8.504)	(10.77)
weektestmay	-13.86	-1.728	-2.935	-7.414
	(15.88)	(11.19)	(8.909)	(12.50)
norepeatersaftertest	0.147	1.207	-5.193	1.701
	(8.610)	(9.087)	(8.834)	(10.24)
weektestmaynoprivate	5.466	1.960	4.400	9.970
	(17.15)	(12.08)	(10.25)	(13.97)
minutsmath		-0.0729***		
		(0.0206)		
minutscie			0.0863***	
			(0.0119)	
minutstotal				-0.00615
				(0.00560)
Constant	295.2***	333.2***	389.5***	405.9***
	(85.65)	(77.14)	(77.91)	(110.2)
Observations	12,264	12,264	12,075	10,092
R-squared	0.290	0.340	0.279	0.244

Standard errors between parentheses.

*** p<0.01

** p<0.05

* p<0.10

## 6. Concluding remarks

This paper examines the Madrid region’s PISA 2018 results to identify the possible causes of the anomalous results in that period. Our belief is that results are affected by two events in the Madrid region: 1) participation in the regional external and standardized test that all students in their final year of compulsory school must take; and 2) the 2017/18 academic calendar change in the region. In both cases, their impact are estimated by using a difference-in-difference approach. We suspect that the regional test affects student performance at PISA because the PISA test took place from April 15th to May 30th, while the regional test came about on April 26th-27th. To identify the effect of this event, we leverage the fact that our control group, grade repeater students, only take the PISA but not the regional assessment. In contrast, non-repeaters (treatment group) take both exams. The analysis includes a set of observable individual, family, and school characteristics that could explain individual differences due to reasons different from treatment. The implicit assumption in our approach is that, once we control for these characteristics, performance differences should be entirely explained by the action of treatment.

We find that taking the PISA test after the regional test in Madrid negatively impacted performance, but the effect is not significant at the conventional values. Additionally, the correlation of PISA scores with exam week in May is minor. Therefore, these results do not suggest that other exams could explain the deficient performance of Madrid students in PISA.

Another possible event that could explain a negative score in the PISA test is its overlap with the exam week in May. However, only students in public and charter schools take these exams while those in private schools take their exams on a different date. We conducted a difference in difference estimation where the students in the control and treatment groups are those who took the PISA test in May and belonged to a non-fee-paying (public and charter) and private school, respectively. As expected, private school students who are non-affected by May exams perform better than their counterparts in non-fee-paying schools. However, the effect is not significant at the conventional values. Conversely, the overlap of the PISA and Madrid exams does not explain the different performance results in repeater and non-repeater students. These results indicate that a crowded schedule resulting from overlaps with other exams has no significant impact on students affected by these events.

It is possible that the reason for the atypical results in Spain is the result of a combination of factors beyond the scope of our measurements. Perhaps these results merit a follow-up study to obtain more significant results as in Brevik and Hellekjær [17].

Potential future research could focus on identifying strategies or interventions that educational institutions can implement to reduce the negative effects of crowded exam schedules. For example, staggered exam schedules or providing additional support during busy testing periods could be explored and the long-term effect of crowded exam schedules on students’ academic performance, motivation, and well-being. Similarly, longitudinal studies could provide insights into how testing schedules may impact students’ educational trajectories. For instance, poor performance because of a crowded schedule may prevent students from obtaining a scholarship and access to higher education. Similarly, low grades may lead to students’ discouragement and lack of interest in continuing their studies.

## Supporting information

S1 Appendix Descriptive statistics for this study. (DOCX)

S1 Data Tables 4–7. (CSV)

S2 Data Values of Fig 1. (CSV)
