# Supplementary material for: The importance of test order in external and standardized test results: The case of PISA 2018
Source: PLoS One. 2024 Sep 9;19(9):e0309980. doi: 10.1371/journal.pone.0309980 (PMC11383243; doi:10.1371/journal.pone.0309980)
Supplement: S1 Appendix — (DOCX) [file pone.0309980.s001.docx]

**S1. Appendix.** **Descriptive statistics of the variables employed in the difference-in-difference models**

| Variable | Obs | Mean | Std. Dev. | Min | Max |  |
| --- | --- | --- | --- | --- | --- | --- |
| female | 16659 | .482 | .5 | 0 | 1 |  |
| age | 16659 | 15.794 | .291 | 15.333 | 18.083 |  |
| inmigrant | 14622 | .108 | .31 | 0 | 1 |  |
| motherinmigrant | 14553 | .208 | .406 | 0 | 1 |  |
| foreignlanguage | 14751 | .054 | .226 | 0 | 1 |  |
| minutsread | 12567 | 231.202 | 71.162 | 0 | 1650 |  |
| minutsmath | 12564 | 233.504 | 73.747 | 0 | 1840 |  |
| minutscie | 12369 | 216.961 | 143.669 | 0 | 1575 |  |
| minutstotal | 10326 | 1770.89 | 410.834 | 120 | 3000 |  |
| ESCS | 14646 | .357 | 1.002 | -3.546 | 3.17 |  |
| biling | 16659 | .227 | .419 | 0 | 1 |  |
| nobiling | 16659 | .231 | .421 | 0 | 1 |  |
| private | 16659 | .207 | .405 | 0 | 1 |  |
| charternobiling | 16659 | .215 | .411 | 0 | 1 |  |
| ESCSschool | 16659 | .329 | .624 | -1.274 | 1.455 |  |
| ESCSschool2 | 16659 | .335 | .647 | -1.624 | 2.118 |  |
| ESCS2 | 14646 | .389 | 1.661 | -12.571 | 10.05 |  |
| DATMadrid | 16659 | .447 | .497 | 0 | 1 |  |
| DATEast | 16659 | .126 | .332 | 0 | 1 |  |
| DATSouth | 16659 | .2 | .4 | 0 | 1 |  |
| DATNorth | 16659 | .076 | .265 | 0 | 1 |  |
| norepeaters | 14757 | .782 | .413 | 0 | 1 |  |
| aftermadridtest | 14904 | .826 | .379 | 0 | 1 |  |
| weektestmay | 14904 | .451 | .498 | 0 | 1 |  |
| norepeatersaftertest | 14709 | .649 | .477 | 0 | 1 |  |
| weektestmaynoprivate | 14904 | .344 | .475 | 0 | 1 |  |
| weektestmay | 14904 | .451 | .498 | 0 | 1 |  |
|  | | | | | | |
